# Indoor Radon Measurements Using Radon Track Detectors and Electret Ionization Chambers in the Bauxite-Bearing Areas of Southern Adamawa, Cameroon

**DOI:** 10.3390/ijerph17186776

**Published:** 2020-09-17

**Authors:** Saïdou, Oumar Bobbo Modibo, Ndjana Nkoulou II Joseph Emmanuel, Olga German, Kountchou Noube Michaux, Hamadou Yerima Abba

**Affiliations:** 1 Nuclear Technology Section, Institute of Geological and Mining Research, Yaounde P.O. Box 4110, Cameroon; bobbomodibo@gmail.com (O.B.M.); nndjana@yahoo.fr (N.N.II.J.E.); kountchounoube@yahoo.fr (K.N.M.); hamadouyerima700@gmail.com (H.Y.A.); 2 Faculty of Science, University of Yaounde I, Yaounde P.O. Box 812, Cameroon; 3 Institute of Radiation Emergency Medicine, Hirosaki University, 66-1 Honcho, Hirosaki-shi, Aomori 036-8564, Japan; 4 Division of Radiation, Transport and Waste Safety, Department of Nuclear Safety and Security, International Atomic Energy Agency, P.O. Box 100, 1400, Wagramer strasse, 1020 Vienna, Austria; O.German@iaea.org

**Keywords:** bauxite, radon, electret ionization chamber, radon track detector, external dose, inhalation dose

## Abstract

The current work deals with indoor radon (^222^Rn) concentrations and ambient dose-equivalent rate measurements in the bauxite-bearing areas of the Adamawa region in Cameroon before mining from 2022. In total, 90 Electret Ionization Chambers (EIC) (commercially, EPERM) and 175 Radon Track Detectors (commercially, RADTRAK^2^) were used to measure ^222^Rn concentrations in dwellings of four localities of the above region. A pocket survey meter (RadEye PRD-ER, Thermo Scientific, Waltham, MA, USA) was used for the ambient dose-equivalent rate measurements. These measurements were followed by calculations of annual doses from inhalation and external exposure. ^222^Rn concentrations were found to vary between 36 ± 8–687 ± 35 Bq m^−3^ with a geometric mean (GM) of 175 ± 16 Bq m^−3^ and 43 ± 12–270 ± 40 Bq m^−3^ with a geometric mean of 101 ± 21 Bq m^−3^ by using EPERM and RADTRAK, respectively. According to RADTRAK data, 51% of dwellings have radon concentrations above the reference level of 100 Bq m^−3^ recommended by the World Health Organization (WHO). The ambient dose equivalent rate ranged between 0.04–0.17 µSv h^−1^ with the average value of 0.08 µSv h^−1^. The inhalation dose and annual external effective dose to the public were assessed and found to vary between 0.8–5 mSv with an average value of 2 mSv and 0.3–1.8 mSv with an average value of 0.7 mSv, respectively. Most of the average values in terms of concentration and radiation dose were found to be above the corresponding world averages given by the United Nations Scientific Commission on the Effects of Atomic Radiation (UNSCEAR). Even though the current exposure of members of the public to natural radiation is not critical, the situation could change abruptly when mining starts.

## 1. Introduction

Mining activities are increasing in Africa. Several countries have recently instituted a mining code to better organize this sector for their socio-economic sustainable development [1]. Like other countries, Cameroon has various mineral ore deposits; however, most of them have not yet been exploited. A national project on strengthening the capacities of the mining sector is being implemented by the Government of Cameroon [2]. The project resulted in new discoveries of more than three hundred anomalies of mineral resources. A large number of mineral resources, such as uranium, thorium, gold, diamond, bauxite, copper, rutile, cobalt, iron, and rare-earth metals, were discovered. Further exploration is required to confirm the occurrence of the ore deposits of the above mineral resources, which would rank Cameroon among the top countries with valuable underground mineral resources. Multiple activities were organized by the Government to promote mining in Cameroon. It is well known that mining leads to environmental pollution by naturally occurring radioactive materials (NORM) and heavy metals. For most human activities involving minerals and raw materials, the levels of exposure to these radionuclides are not significantly greater than normal background levels, and when these raw materials with low concentrations of NORM are introduced in an industrial process, the radionuclides can become more concentrated in the produced by-products or in waste material such as red mud in bauxite mining [3,4]. Such activities significantly increase the exposure of workers and the general public, meaning that they may need to be controlled by regulation in order to ensure the protection from radiation of relevant people. The most frequently-occurring radionuclides and their decay products found in bauxite and bauxite processing residuals include ^238^U, ^235^U, and ^232^Th series.

Bauxite ore is the primary source of aluminum in the world [5]. The ore must first be chemically processed to produce alumina (aluminum oxide), which is then smelted using an electrolysis process to produce pure aluminum metal. Bauxite is typically found in topsoil located in various tropical and subtropical regions. Bauxite reserves are most plentiful in Africa, Oceania and South America. Reserves are projected to last for centuries. Bauxite is a rock formed from a reddish clay material called laterite soil and is most commonly found in tropical or subtropical regions. Bauxite is primarily comprised of aluminum oxide compounds (alumina), silica, iron oxides and titanium dioxide. More than 160 million metric tons of bauxite are mined each year. The leaders in bauxite production include Australia, China, Brazil, India and Guinea. Bauxite reserves are estimated to be 55 to 75 billion metric tons, primarily spread across Africa (32%), Oceania (23%), South America and the Caribbean (21%) and Asia (18%). 

The bauxite deposits of Minim-Martap and Ngaoundal in southern Adamawa, Cameroon will be exploited in the near future. Thus, pre-exploitation background radiation levels should be determined for the accurate assessment of the impact of mining on the environment and the general public in the post-exploitation environment [6,7,8,9,10,11,12,13,14,15]. In that perspective, field works were performed for soil and water sampling, radon detectors were deployed in dwellings and ambient equivalent dose rates measurements were performed. In this work, the results on radon measurements in 265 dwellings using two types of detectors (EPERM and RADTRAK), ambient-equivalent dose rates measurements both indoors and outdoors followed by inhalation and external dose assessments are reported. Additionally, the limitations of EPERM detectors compared to RADTRAK detectors are discussed. 

## 2. Materials and Methods 

### 2.1. Study Areas

**Figure 1:**
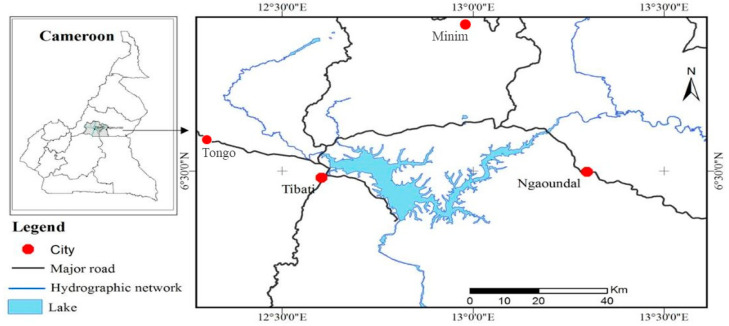
Location map of the bauxite bearing areas of southern Adamawa.

The study areas are located in Djerem Division, in the southern part of Adamawa Region in Cameroon (Figure 1). The Division has about 200,000 inhabitants, with Tibati as the capital. Several bauxite deposits are located particularly in Ngaoundal and Minim-Martap. The region is mountainous and forms the barrier between the forest area of south Cameroon and the savannah area of North Cameroon. The climate is typically tropical Sudanese with two seasons: the dry season from November to April and rainy season from May to October. The annual rainfall varies between 900 mm to 1500 mm. Temperatures vary between 10.1 to 41.6 °C, and the relative humidity varies between 10.2–99.9% throughout the year [16]. 

Canyon Resources is an Australian mining company which has exploration and mining permits covering several bauxite deposits in Cameroon, including in the Adamawa Region—the present study area [17]. Its local branch, Camalco SA, is focused on developing the Minim-Martap Bauxite Project (MMP). The MMP has identified a bauxite-rich plateau which was mapped after drilling and prospection across the surface. The total area covered by the permits is 1349 km^2^. The bauxite is generally high in alumina, with low total and reactive silica, high gibbsite, low boehmite and low amounts of other contaminants [18]. The total indicated resources are 839 Mt at 45.2% of Al_2_O_3_ and 2.8% of SiO_2_. The bauxite also includes a high-grade (cut-off grade 45% Al_2_O_3_) indicated resource of 431 Mt at 48.8% of Al_2_O_3_ and 2.6% of SiO_2_ containing substantial zones of >50% of Al_2_O_3_ with very low amounts of contaminants [17].

### 2.2. Methodology

#### 2.2.1. Electret Ionization Chamber

E-PERM Electret Ion Chambers (EICs) were used to measure ^222^Rn indoors. The E-PERM EICs were manufactured by Rad Elec Inc., Frederick, MD, USA. Detailed descriptions of their design and operation have been given in the Rad Elec Manual and also published in [19]. An EIC for monitoring radon consists of a stable electret (electrically charged Teflon^®^ disc) mounted inside an electrically conducting chamber. The electret serves both as a source of the electric field and as a sensor. The ions produced inside the chamber are collected by the electret. The reduction in charge of the electret is related to the total ionization during the period of exposure. This charge reduction is measured using a battery-operated Electret Voltage Reader. Using appropriate calibration factors and exposure time, desired parameters such as airborne radon concentration in the air can be calculated. 

Ninety EIC type LLT (Low Sensitivity L+ Electret Long Term LT) devices were randomly and equally distributed in Ngaoundal and Tibati—the most populated towns of Djerem Division, representing about 50% of people living in the Division. About 40,000 and 60,000 inhabitants live in Ngaoundal and Tibati, respectively. The exposure period was from December 2014 to February 2015 during the dry season. Although indoor radon concentrations are submitted to seasonal variations, no correction was carried out. Dwellings were selected randomly with an in-situ request addressed to the resident to place an EIC inside the house. EICs were exposed for two months relatively far from the open access of house to avoid biased measurements due to the influence of outdoor air and at 1 m above the ground. Most of the surveyed dwellings were built using locally made soil bricks, which were sometimes covered by a thick layer of cement. 

Radon concentrations were given by the following equation:

(1) $$C_{Rn}\left(Bq\;m^{-3}\right)=37\times \left(\frac{I-F}{CF.D}-BG\right)f_{corr}^{att}\left(pCi\;L^{-1}\right)\;CF=A+B\frac{I+F}{2}$$

where *I* and *F* are the initial and final voltages of the electret expressed in volts [V], *CF* is the calibration factor [V/pCi L^−1^ days], *D* is the duration of the exposure [days], *BG* is the background due to the ambient dose expressed in radon equivalent concentration [pCi L^−1^] and $f_{corr}^{att}$ is the correction factor taking into account of the dwelling altitude (alt) above sea level. The fitting parameters *A* and *B* are given by the manufacturer as *A* = 0.02383 and *B* = 0.0000112.

(2) $$BG=0.120\frac{pCi\;L^{-1}}{\mu R\;h^{-1}}\;f_{corr}^{att}=0.996+0.00016.\mathrm{alt}\left(m\right)$$

EICs are sensitive to background gamma radiation. The equivalent radon signal in picocuries per liter (pCi L^−1^) per unit background radiation in micro-roentgens per hour (µR h^−1^) is determined by the manufacturer depending on the type of EIC. This is specific to the chamber and not to the electret used in the chamber. This parameter is 0.12 for L chambers. This value must be multiplied by the gamma radiation level at the site (in µR h^−1^) and the product (in equivalent pCi L^−1^) subtracted from the apparent radon concentration. The default value of the background was used for the present study.

The minimum voltage before exposing the EIC is fixed at 200 V. The accuracy of measurements is ensured by using a radon reference chamber for quality control checking before each set of electret readings using the Electret Voltage Reader. The voltage of the reference chamber when provided by the manufacturer is (248 ± 1) V. This voltage is supposed to decrease less than 1 V each year and should not be used for many years.

The precision was monitored in the present study by placing 10 EICs in the same conditions in a dwelling for three months. The arithmetic mean of the indoor radon concentration and the standard deviation were determined. The corresponding precision was determined to be 10% [6].

Regarding the uncertainty assessment, three sources of uncertainty were identified: (1) uncertainty *u*_1_, regarding the active volume and electret thickness of the EIC estimated at 5%; (2) uncertainty *u*_2_, related to the initial and final readings of the electret estimated at 1.4 V; (3) uncertainty *u*_3_, regarding the gamma external radiation, estimated between 0.1–0.2 pCi L^−1^. The uncertainties of the temperature, humidity and ventilation system were neglected. 

Finally, the overall relative uncertainty combining all the above components was evaluated by using the following equation:

(3) $$u=\sqrt{u_{1}^{2}+u_{2}^{2}+u_{3}^{2}}$$

#### 2.2.2. Closed Alpha-Track Detector (RADTRAK²^®^)

A number of 175 RADTRAK detectors were deployed in Ngaoundal, Minim, Tibati and Tongo from May to June 2019. A total of 169 detectors were collected after two months of exposure time and sent back to the RADONOVA Laboratories for analysis in Uppsala, Sweden. The measurement was performed following the standard ISO 11665-4 [20]. The detector container was manufactured from electrically conducting plastic. Through a small slit (filter), radon gas entered the detector. The track-detecting material (film) inside the detector is was then by alpha particles generated by the radon entering the container and the decay products formed from it. On the film, the alpha particles made small tracks which are enlarged with chemical etching and later counted in a microscope in order to determine the radon exposure. The lowest detection limit for a measurement period of 3 months is 10 Bq m^−^³. 

The arithmetic mean of radon activity concentration (Bq m^−3^) is given as follows:

(4) $$\bar{C}=\left(n_{g}-{\bar{n}}_{b}\right)\frac{1}{t.S_{SSNTD}.F_{c}}=\left(n_{g}-{\bar{n}}_{b}\right).\omega \;\omega =\frac{1}{t.S_{SSNTD}.F_{c}}$$

where $n_{g}$ is the number of tracks after exposure, ${\bar{n}}_{b}$ is the mean number of tracks caused by the background radiation, *t* is the sampling duration, $F_{c}$ is the calibration factor, ω is the correction factor linked to the calibration factor and the sampling duration and $S_{SSNTD}$ is the detector area used for counting the number of etched tracks in cm^2^.

For the most accurate value, ${\bar{n}}_{b}$ is determined experimentally by reading *n* detectors that have not been exposed to radon and have been processed under the same physico-chemical and counting conditions. The value of ${\bar{n}}_{b}$ may also be given by the manufacturer.

The standard uncertainty of $\bar{C}$ is given as follows:

(5) $$u\left(\bar{C}\right)=\sqrt{\left(n_{g}+\frac{{\bar{n}}_{b}}{n}\right).\omega^{2}+{\bar{C}}^{2}.u_{rel}^{2}\left(\omega \right)}\;u_{rel}^{2}\left(\omega \right)=u_{rel}^{2}\left(F_{c}\right)+u_{rel}^{2}\left(S\right)$$

where $u_{rel}$ is the relative standard uncertainty. The uncertainty of the sampling duration is considered negligible.

#### 2.2.3. Inhalation Dose

The inhalation dose due to the exposure to radon is given as follows:

(6) $$E_{inh}=A_{inh}\times e_{inh}\times F_{occ}\times F_{eq}\times t$$

where $A_{inh}$ is the geometric mean of radon concentration, $e_{inh}$ is the inhalation dose conversion factor of 9 nSv/(Bq h m^−3^), $F_{occ}$ is the occupancy factor of 0.6 for the study areas, $F_{eq}$ is the equilibrium factor of 0.4 which is the default value given by UNSCEAR [21] and *t* corresponds to one year expressed in hours.

It should be mentioned that the conversion factor proposed by UNSCEAR [21] has recently been called into question by the International Commission on Radiological Protection (ICRP) [22], which suggests a correction by a factor of 2 upwards. In 2017, the ICRP published new, higher dose conversion factors for radon, which therefore increased the calculated radiation dose associated with exposure to radon in workplaces [23]. For the calculation of doses following the inhalation of radon and radon progeny in underground mines and in buildings, in most circumstances, the ICRP recommends a dose coefficient of 3 mSv per mJ h m^−3^ (approximately 10 mSv WLM^−1^).

UNSCEAR, however, has confirmed in a report on lung cancer from exposure to radon in 2019 that the evidence reviewed by its experts is compatible with the available data in the Committee’s previous assessment of lung cancer risk due to radon [24]. Therefore, UNSCEAR concluded that there is no reason to change its established dose conversion factor and recommends the continued use of the dose conversion factor of 9 nSv per (Bq h m^−3^) EEC of ^222^Rn, which corresponds to 1.6 mSv (mJ h m^−3^)^−1^ for estimating radon exposure levels to a population. This new report was approved by the Fourth Committee of the United Nations General Assembly in October 2019.

Finally, it is clear that the uncertainty in the dose conversion factor should be taken into consideration when assessing inhalation dose due to radon indoors.

#### 2.2.4. External Effective Dose

The Thermo Scientific RadEye PRD detector was used to measure the ambient dose-equivalent rates prior to external dose assessment. It is a high-sensitivity gamma radiation detection and dose rate measurement tool incorporating a highly sensitive NaI(TI) scintillation detector with a miniature photomultiplier for the detection of gamma radiation levels.

The radiation exposure from external sources is the result of natural and artificial ground radiation as well as from the cosmic background. The value of the external effective dose is given by

(7) $$E_{ext}\;\left(mSv\right)=\left[\left(1-F_{occ}\right)H_{out}+F_{occ}\times H_{in}\right]\times t$$

where $H_{out}$ and $H_{in}$ are the arithmetic mean of the outdoor and indoor ambient equivalent dose rates and $F_{occ}$ is the occupancy factor of 0.6 determined previously by Saïdou et al. [6].

## 3. Results and Discussion

### 3.1. Indoor Radon Distribution Using Electret Ionization Chambers (EPERM)

**Figure 2:**
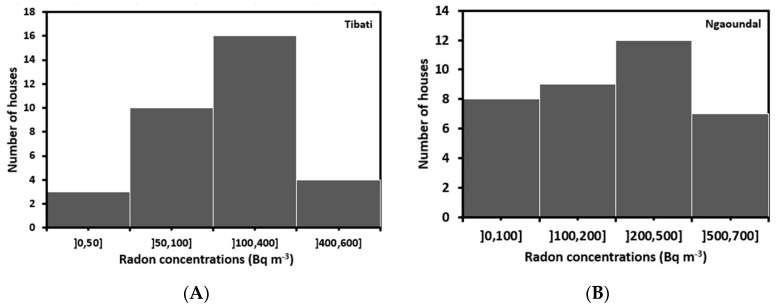
Distribution of radon in dwellings of Tibati (**A**) and Ngaoundal (**B**) using EPERM detectors.

**Figure 3:**
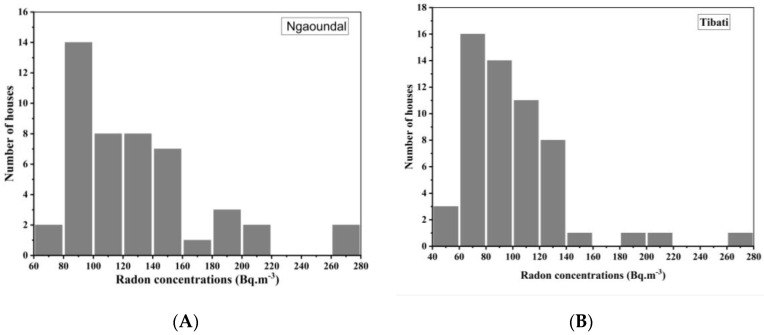
Lognormal distribution of ^222^Rn in houses of Ngaoundal (**A**) and Tibati (**B**) using RADTRAK detectors. The numbers of houses are 48 and 56 in Ngaoundal and Tibati, respectively.

From the total of 90 EPERM detectors deployed, 69 were returned in a usable condition in laboratory for the final voltage reading and radon concentration deduction. Figure 2 and Figure 3 display the distributions of indoor radon concentrations in Tibati and Ngaoundal, respectively. The minimum and maximum radon concentrations were 36 Bq.m^−3^ and 569 Bq m^−3^ in Tibati, 37 Bq m^−3^ and 687 Bq m^−3^ in Ngaoundal respectively. The corresponding arithmetic and geometric means were 195 Bq m^−3^ and 150 Bq m^−3^ in Tibati, 270 Bq m^−3^ and 205 Bq m^−3^ in Ngaoundal, respectively. These values are clearly above the world arithmetic mean of 40 Bq m^−3^ [21]. It has been proven that elevated radon concentrations indoors depend on several factors such as the building material, radon exhalation rate from the ground and house ventilation rate [25,26,27,28]. In most surveyed dwellings, the buildings were poorly ventilated and were made out of soil bricks. Such conditions could explain the high indoor radon concentrations. In its recent Publication 126 [29], the International Commission on Radiological Protection (ICRP) strongly encouraged national authorities to set national reference levels in the range of 100–300 Bq m^−3^ by taking into account the socio-economic factors of the country. It was found that 70% and 15% of surveyed dwellings had radon concentrations above 100 Bq m^−3^ and 300 Bq m^−3^, respectively. In view of the latest scientific data, the WHO proposed a reference level of 100 Bq m^−3^ to minimize health hazards due to indoor radon exposure [30]. If this level cannot be reached by taking into account the Gross Domestic Product (GDP) of the country, the chosen reference level should not exceed 300 Bq m^−3^ in houses. 

### 3.2. Indoor Radon Distribution Using Radon Track Detectors (RADTRAK)

**Figure 4:**
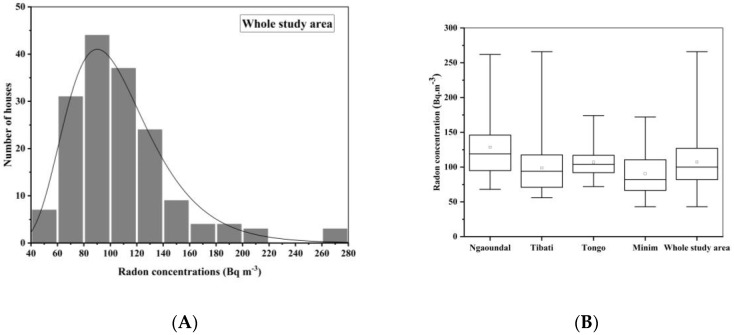
Lognormal distribution (**A**) of indoor ^222^Rn in the bauxite-bearing Southern Adamawa. Boxplot distribution (**B**) is made for each study area and the whole bauxite-bearing areas. The total number of RADTRAK detectors analyzed was 169. Box plot refers to median, lower and upper quartiles. Outliers are shown on either side of the rectangular box at the limit of the vertical line. The small filled circle above the median represents the arithmetic mean.

As shown in Table 1, ^222^Rn concentrations were found to vary between 43 ± 12 and 270 ± 40 Bq m^−3^ with a geometric mean of 102 ± 21 Bq m^−3^. It was also found that 51% of dwellings had radon concentrations above the reference level of 100 Bq m^−3^ recommended by the WHO [21]. About 4% of dwellings had radon concentrations higher than 200 Bq m^−3^. No dwelling had a radon concentration above 300 Bq m^−3^. Radon concentrations in Ngaoundal and Minim ranged between 68 ± 14 and 262 ± 40 Bq m^−3^ and 43 ± 12 and 172 ± 30 Bq m^−3^, respectively, with corresponding GMs of 123 ± 24 Bq m^−3^ and 85 ± 18 Bq m^−3^, respectively. Radon concentrations in Tibati and Tongo ranged between 56 ± 14 and 270 ± 40 Bq m^−3^ and 72 ± 14 and 174 ± 30 Bq m^−3^, respectively, with corresponding GMs of 93 ± 20 Bq m^−3^ and 105 ± 21 Bq m^−3^. As shown in Figure 4 radon distribution in the whole study area follows lognormal law.

The above values were compared with other results from ore-bearing or mining regions of Cameroon [6,7,8,9,10,11,12,13,14,15]. Indoor radon concentrations ranged between 46–143 Bq m^−3^ in Poli [12], 27–937 Bq m^−3^ in Lolodorf [15], 88–282 Bq m^−3^ in Betare-Oya [9], and 31–436 Bq m^−3^ in the coastal city of Douala [11]. The average values were 82 Bq m^−3^, 97 Bq m^−3^, 133 Bq m^−3^ and 139 Bq m^−3^, respectively. In total, 20% of dwellings had radon concentrations above 100 Bq m^−3^ in the uranium-bearing region of Poli, 47% in the uranium-bearing region of Lolodorf, 76% in the gold mining areas of Betare-Oya and 27% in Douala city. No house had a radon concentration above 300 Bq m^−3^ in Poli and Douala city. Only 1% of houses had a radon concentration above 300 Bq m^−3^ in Lolodorf, and 3% in Betare-Oya. These results indicate a large variation in the radon level in houses. It is clear that the above data make a real contribution to radon-risk mapping in the country.

**Figure 5:**
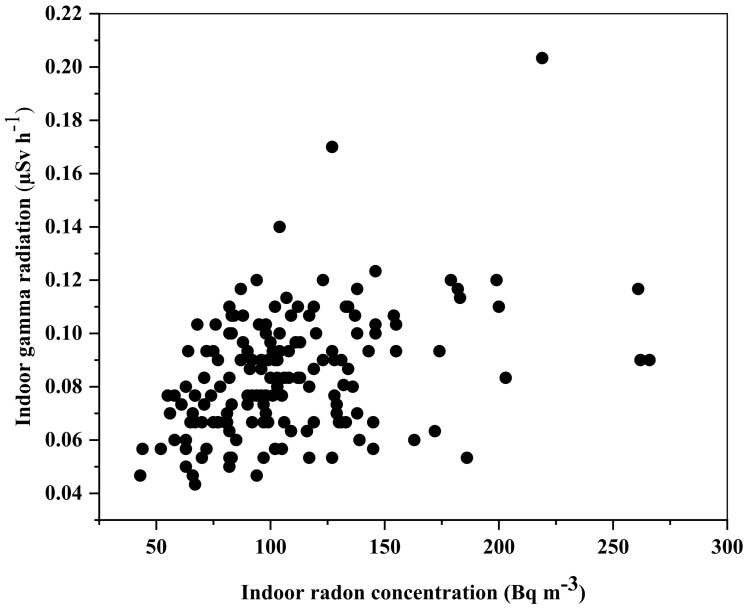
Correlation between indoor ambient-equivalent dose rate and indoor radon in the bauxite-bearing areas of southern Adamawa.

As evidenced in Figure 5, the Pearson correlation factor of 0.4 shows that the correlation between the indoor ambient-equivalent dose rate and ^222^Rn concentration is low in the bauxite-bearing areas of southern Adamawa in Cameroon. This low correlation reveals an independence between indoor gamma radiation and radon concentration due to the good ventilation of dwellings. Doors and windows are diurnally open.

### 3.3. Comparison of the Results Obtained with EPERM and RADTRAK 

Wide differences were observed between results given by EPERM and RADTRAK detectors. Table 2 shows the coefficient of the ratio between EPERM and RADTRAK, which varied greatly from 0.80 to 2.50. Moreover, the Z-score test on the two sets of data confirmed the wide difference between most of the values belonging to the two sets of data (EPERM and RADTRAK). EPERM is a detector based on the principle of electrostatic collection; it has been shown that this type of detector is greatly affected by humidity, such as with the Electrostatic Radon Monitor developed by Iida et al. [31]. Furthermore, Sorimachi et al. [32] showed how humidity, ambient aerosols and thoron influence the detection responses of EIC detectors. The present study area is a humid climate area with a yearly humidity ranging between 10–99% [18]. This could explain the great difference between the two sets of data. Furthermore, the dwellings surveyed by EPERM detectors in 2015 are not in general those where ^222^Rn was measured in 2019 using RADTRAK detectors.

### 3.4. External Effective Dose

Table 3 shows that the external effective dose in the studied areas of Ngaoundal, Minim, Tongo and Tibati ranges between 0.3–1.8 mSv with an arithmetic mean of 0.7 ± 0.2 mSv. It ranges between 0.6–1.8 mSv in Ngaoundal, 0.3–0.7 mSv in Minim, 0.5–0.8 mSv in Tibati and 0.6–0.9 mSv in Tongo. The corresponding arithmetic means are 0.9 ± 0.2 mSv, 0.5 ± 0.1 mSv, 0.6 ± 0.1 mSv and 0.8 ± 0.1 mSv, respectively. Most of these values are lower than the world average value of 0.9 mSv (0.5 mSv for terrestrial radiation and 0.4 mSv for cosmic radiation) given by UNSCEAR [21]. Currently, the exposure of members of the public to external sources is not critical. The situation could change in Ngaoundal and Minim, which are most close to the bauxite deposits. According to Canyon Resources, mining will start in 2022, with a consequent increase in the external dose due to NORM.

### 3.5. Inhalation Dose

**Figure 6:**
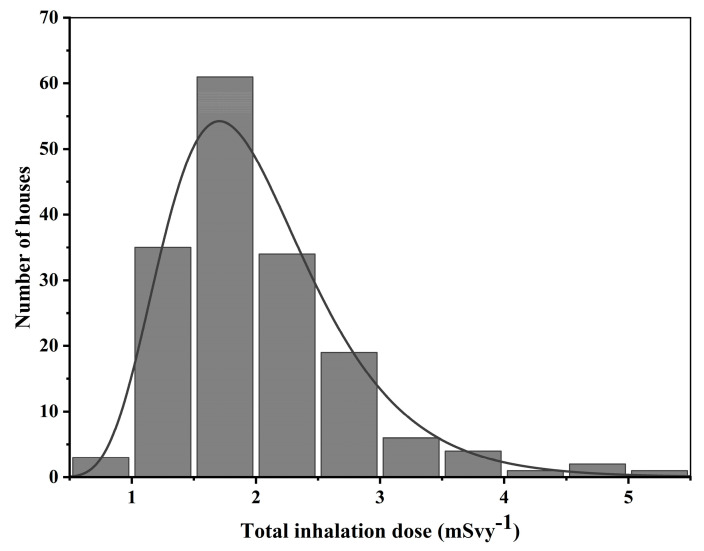
Lognormal distribution of inhalation dose due to ^222^Rn in dwellings in the bauxite-bearing areas of Southern Adamawa.

As displayed in Table 3, the inhalation dose of the studied areas ranged between 0.8–5 mSv with an average value of 2.0 ± 0.7 mSv. It ranged between 1.3–5 mSv, 0.8–3.3 mSv, 1.1–5 mSv and 1.4–3.3 mSv in Ngaoundal, Minim, Tibati and Tongo. respectively. The corresponding average values are 2.4 ± 0.9 mSv, 1.7 ± 0.6 mSv, 1.9 ± 0.7 mSv and 2.0 ± 0.4 mSv, respectively. All the average values were higher than the world average value of 1.2 mSv given by UNSCEAR [21]. Saïdou et al. [10] previously reported natural radiation exposure to the public in some mining and ore-bearing regions of Cameroon. It has been found that the average inhalation doses due to ^222^Rn are 1.5 mSv, 2 mSv, 2.5 mSv and 2.6 mSv in the uranium-bearing region of Poli [12], the uranium and thorium-bearing region Lolodorf [15], the gold mining areas of Betare-Oya [9] and the coastal city of Douala [11], respectively. It can be noted that the above average values are comparable to those found in the current work. As shown in Figure 6, the inhalation dose distribution of indoor radon follows the lognormal law.

## 4. Conclusions

In this work, ^222^Rn was measured in 265 dwellings of the bauxite-bearing areas of southern Adamawa in Cameroon using EPERM and RADTRAK detectors followed by indoor and outdoor ambient equivalent dose rate measurements before the commencement of mining in 2022. The ^222^Rn concentrations were found to vary between 43–270 Bq m^–3^, with a geometric mean of 101 Bq m^−3^—higher than the world average value of 40 Bq m^−3^. It was also found that 51% of dwellings have radon concentrations above the reference level of 100 Bq m^−3^. The inhalation dose due to ^222^Rn exposure ranges between 0.8–5 mSv with an average value of 2 mSv—higher than world average value of 1.2 mSv. The external effective dose ranges between 0.3–1.8 mSv with an average value of 0.7 mSv—lower than the world average value of 0.9 mSv (0.5 mSv for terrestrial radiation and 0.4 mSv for cosmic radiation) given by UNSCEAR. It clearly appears that the exposure level is not critical. The situation could change in the near future during mining activity. Thus, radiation protection measures should be applied to avoid the spread of NORM in the environment. Furthermore, the current results will contribute in the ongoing project of establishing national reference levels for indoor radon in Cameroon within the framework of the setup of the National Radon Action Plan and regulations of radon exposure. 

## Figures and Tables

**Table 1:** Activity concentrations of ^222^Rn determined by using Radon Track (RADTRAK) detectors. AM: arithmetic mean, SD: standard deviation, GM: geometric mean, GSD: geometric standard deviation, N: number of dwellings.

Statistical Parameters	Radon Concentration (Bq m^−3^)				
	Ngaoundal	Tibati	Tongo	Minim	Whole Study Area
Range	68–262	56–270	72–174	43–172	43–270
AM ± SD	131 ± 25	99 ± 21	107 ± 22	91 ± 19	108 ± 22
GM ± GSD	123 ± 24	93 ± 20	105 ± 21	85 ± 18	102 ± 21
Median	123	94	104	82	100
N	48	56	31	32	167

**Table 2:** Ratio (RADTRAK/ EPERM) activity concentrations of indoor ^222^Rn and Z-score test.

^222^Rn Concentration (Bq m^−3^) by EPERM	^222^Rn Concentration (Bq m^−3^) by RADTRAK	Type	Ratio (RADTRAK/EPERM	Z-Score
36 ± 8	43 ± 12	Minimum	0.80	−0.48
687 ± 35	270 ± 40	Maximum	2.50	7.8
233 ± 20	108 ± 22	Arithmetic mean	2.20	4.23
175 ± 16	101 ± 21	Geometric mean	1.70	2.8

**Table 3:** External and inhalation dose to members of the public in the bauxite-bearing areas of Southern Adamawa. AM: arithmetic mean, SD: standard deviation, GM: geometric mean, GSD: geometric standard deviation, N: number of dwellings.

	Statistical Parameters	External Effective Dose (mSv)			Inhalation Dose (mSv)
		Outdoor (Out)	Indoor (In)	Total Dose (Out + In)	
Ngaoundal	Range	0.20–0.70	0.39–1.07	0.64–1.77	1.29–4.96
	AM ± SD	0.31 ± 0.08	0.56 ± 0.12	0.86 ± 0.18	2.43 ± 0.87
	GM(GSD)	0.30 ± 1.24	0.54 ± 1.20	0.85 ± 1.18	2.30 ± 1.38
	Median	0.30	0.53	0.82	2.26
	*N*	48	48	48	48
Tibati	Range	0.18–0.30	0.28–0.54	0.50–0.84	1.06–5.04
	AM ± SD	0.23 ± 0.03	0.39 ± 0.05	0.62 ± 0.06	1.87 ± 0.72
	GM(GSD)	0.23 ± 1.14	0.39 ± 1.14	0.62 ± 1.12	1.77 ± 1.39
	Median	0.23	0.38	0.62	1.78
	*N*	56	56	56	56
Tongo	Range	0.21–0.35	0.35–0.63	0.63–0.94	1.36–3.30
	AM ± SD	0.27 ± 0.03	0.50 ± 0.07	0.77 ± 0.08	2.03 ± 0.40
	GM(GSD)	0.27 ± 1.13	0.49 ± 1.16	0.77 ± 1.10	1.99 ± 1.21
	Median	0.28	0.49	0.78	1.97
	*N*	31	31	31	31
Minim	Range	0.07–0.26	0.23–0.44	0.30–0.68	0.81–3.26
	AM ± SD	0.17 ± 0.03	0.31 ± 0.05	0.48 ± 0.07	1.72 ± 0.62
	GM(GSD)	0.17 ± 1.26	0.31 ± 1.16	0.48 ± 1.17	1.62 ± 1.43
	Median	0.18	0.30	0.48	1.55
	*N*	32	32	32	32
Whole study area	Range	0.07–0.70	0.23–1.70	0.30–1.77	0.81–5.04
	AM ± SD	0.25 ± 0.07	0.44 ± 0.11	0.70 ± 0.18	2.03 ± 0.75
	GM(GSD)	0.24 ± 1.32	0.43 ± 1.30	0.67 ± 1.28	1.92 ± 1.40
	Median	0.25	0.43	0.68	1.90
	*N*	166	166	166	166
